# Transformational Leadership and Heterogeneity in Teachers’ Readiness for AI Integration: A Complementary Variable- and Person-Centered Approach

**DOI:** 10.3390/bs16101892

**Published:** 2026-10-13

**Authors:** Yaxuan Geng, Kangjie Geng, Kai Wang, Qianqian Ruan

**Affiliations:** 1 School of Education, Minzu University of China, Beijing 100081, China; 24400217@muc.edu.cn; 2 Faculty of Education, Beijing Normal University, Beijing 100875, China; 3 Center for Teacher Education Research, Beijing Normal University, Beijing 100875, China; wangkai1991@bnu.edu.cn; 4 Educational Administration and Policy, Faculty of Education, The Chinese University of Hong Kong, Hong Kong, China; ruanqianqian@link.cuhk.edu.hk

**Keywords:** AI tools, behavioral intention, readiness for AI integration, UTAUT2, self-determination theory

## Abstract

Artificial intelligence (AI) in education has attracted increasing global attention. Existing studies have mainly examined teachers’ AI adoption intention in relation to technology-related perceptions and broader school-level conditions, but the role of transformational leadership remains underexplored. Drawing on complementary perspectives from the Unified Theory of Acceptance and Use of Technology 2 (UTAUT2) and Self-Determination Theory (SDT), this study examined transformational leadership in relation to teachers’ readiness for AI integration using complementary variable- and person-centered analyses of data from 596 Chinese in-service K-12 teachers. The variable-centered results showed that transformational leadership was positively associated with teachers’ behavioral intention to use AI tools, with indirect associations involving willingness to develop AI competence and constructivist teaching beliefs. The person-centered analysis identified four readiness profiles: (1) Favorable Orientation but Behavioral Hesitation, (2) Limited Orientation and Behavioral Hesitation, (3) Low Orientation and Weak Behavioral Inclination, and (4) Favorable Orientation and Behavioral Inclination. Profiles 1 and 4 both showed relatively high constructivist beliefs and willingness to develop AI competence, but Profile 4 reported stronger behavioral intention to use AI tools. Profiles 2 and 3 were below the sample average across all three indicators, with Profile 3 showing the lowest behavioral intention. Relative to Profile 1, higher transformational leadership was associated with greater odds of membership in Profile 4 and lower odds of membership in Profile 3. These findings suggest that teachers’ readiness for AI integration varies in both level and configuration, highlighting the need for differentiated leadership and professional support.

## 1. Introduction

The rapid advancement of artificial intelligence (AI) is transforming educational practices in multiple ways. From intelligent tutoring systems to generative content tools, AI technologies are increasingly being integrated into curriculum design, instructional delivery, assessment, and educational management (Holmes et al., 2019; Hadi Mogavi et al., 2024). Within this evolving landscape, teachers play a central role in determining how AI is interpreted, adapted, and implemented in classroom settings. Consequently, teachers’ behavioral intention to engage with and use educational AI in teaching (BI) has become an important consideration in understanding the integration of AI into educational practice. Existing evidence suggests that the availability of technological resources alone may not guarantee successful implementation if teachers remain hesitant to incorporate AI into their teaching (Kong et al., 2024; Wu et al., 2025). Understanding the factors associated with BI therefore remains an important issue in educational technology research.

Research on teachers’ AI adoption has primarily focused on technology-related perceptions, including perceived usefulness, perceived ease of use, self-efficacy, and attitudes, as correlates of teachers’ behavioral intention to use AI-based or generative AI tools (Kong et al., 2024; Wang et al., 2021). Recent multilevel evidence has extended this perspective by showing that school-level conditions, such as technological infrastructure, ICT leadership, shared school vision, and school-provided training, are also associated with teachers’ behavioral intention to use AI (Wu et al., 2025). However, evidence remains limited regarding how transformational leadership is associated with teachers’ AI adoption intentions in relation to their pedagogical beliefs and willingness to develop AI competence.

Transformational leadership (TL) has been widely recognized as an important organizational factor in educational change. By articulating a shared vision, providing individualized support, and encouraging professional growth, transformational leaders may help create organizational conditions that support innovation and adaptation (Leithwood & Jantzi, 2005). Such support may be particularly relevant in the context of AI adoption, where technological capabilities, pedagogical applications, and institutional expectations continue to evolve. Alongside the leadership context, teachers’ pedagogical beliefs may shape how they interpret the instructional value of AI. Constructivist teaching beliefs (CT) emphasize learner-centered instruction, active knowledge construction, and meaningful classroom interaction, which may be particularly relevant when AI is used to support learner-centered and interactive forms of instruction. Previous research has shown that teachers holding stronger CT are generally more receptive to student-centered approaches to technology integration (Ertmer & Ottenbreit-Leftwich, 2010; Petko, 2012). In addition, because AI integration requires teachers to continually update their knowledge and skills, teachers’ willingness to develop AI competence (TW) may be another relevant factor. TW reflects teachers’ readiness to engage in AI-related professional learning and competence development. Although recent studies have highlighted the importance of AI literacy and professional learning (Kong et al., 2024), relatively little is known about how TL, CT, and TW are jointly associated with BI.

A related methodological issue is that much of the existing research has relied on variable-centered approaches. Studies have commonly used structural equation modeling and other variable-centered approaches to examine relationships among variables (Hu et al., 2025; Kong et al., 2024; Wu et al., 2025). While these approaches provide important information regarding relationships among variables across the sample, they may overlook meaningful heterogeneity among teachers. Person-centered research has demonstrated that teachers differ substantially in their motivations for technology use and in their broader adoption patterns (Wu et al., 2021; Xu, 2022). However, little is known about whether distinct configurations of pedagogical beliefs, willingness to develop AI competence, and behavioral intention exist among teachers in the context of educational AI. From a person-centered perspective, the joint configuration of CT, TW, and BI may characterize different patterns of teachers’ readiness for AI integration. Identifying such profiles may provide a more nuanced understanding of teachers’ readiness for AI integration and help inform more targeted support strategies. Furthermore, TL may be differentially associated with membership in these profiles.

Against this background, the present study draws on complementary perspectives from UTAUT2 and SDT and adopts both variable- and person-centered analyses. Structural equation modeling was used to examine the associations among TL, CT, TW, and BI. Latent profile analysis was subsequently conducted to identify distinct teacher profiles based on CT, TW, and BI, followed by an examination of whether TL was associated with profile membership. This design allows the study to examine both associations among TL, CT, TW, and BI across the sample and heterogeneous readiness profiles across teachers. Accordingly, the following research questions were proposed:

RQ1: How is TL associated with BI, and what roles do CT and TW play in these relationships?

RQ2: What latent profiles can be identified based on CT, TW, and BI?

RQ3: Is TL associated with teachers’ latent profile membership?

## 2. Literature Review

### 2.1. AI Integration in Education and Teachers’ Adoption

Recent advances in artificial intelligence (AI), particularly generative AI (GenAI), have attracted considerable attention in educational research. GenAI tools have been increasingly discussed as supports for content generation, feedback, assessment, classroom support, and adaptive learning (Jauhiainen & Garagorry Guerra, 2023; Kasneci et al., 2023; Hadi Mogavi et al., 2024; Yan et al., 2024). These developments have also been accompanied by policy initiatives in several countries that encourage the integration of AI technologies into teaching and learning practices (Ministry of Education of the People’s Republic of China, 2024; Office of Educational Technology, U.S. Department of Education, 2024).

As AI tools become more accessible in educational contexts, teachers’ acceptance and intended use of these technologies have become central issues in AI adoption research. Existing studies have examined educators’ perceptions of GenAI, including perceived instructional opportunities, ethical concerns, and conditions for responsible implementation (Hadi Mogavi et al., 2024). Quantitative studies have largely drawn on the Technology Acceptance Model (TAM), UTAUT2, and related frameworks to examine the associations of perceived usefulness, perceived ease of use, self-efficacy, effort expectancy, social influence, and attitudes with behavioral intention (Hu et al., 2025; Kong et al., 2024; Wang et al., 2021). Among Chinese pre-service teachers, effort expectancy, social influence, hedonic motivation, and habit have been identified as significant correlates of behavioral intention to use GenAI in future teaching within the UTAUT2 framework (Hu et al., 2025). Among elementary school teachers, AI self-efficacy has also been shown to be directly associated with behavioral intention to use AI in education, with perceived usefulness and perceived ease of use serving as mediating factors (Hosseini, 2025). However, existing research has focused primarily on teachers’ individual cognitive evaluations of AI technologies, with comparatively less attention to organizational support, pedagogical beliefs, and motivation for AI-related professional development.

### 2.2. Transformational Leadership, Constructivist Teaching Beliefs, and Willingness to Develop AI Competence

Transformational leadership (TL) reflects leadership practices within the school context that may be particularly relevant when institutional guidelines for AI integration remain underdeveloped. Prior research has shown that school-level conditions, such as ICT resources, ICT leadership, alignment between teachers and schools, and school-provided ICT training, may be directly or indirectly associated with teachers’ behavioral intention to use AI (Wu et al., 2025). However, these factors have often been examined through broader contextual indicators, leaving the role of specific leadership approaches less clear. In the context of AI integration, this issue is particularly important because teachers are required to navigate evolving technological capabilities, pedagogical applications, and institutional expectations. TL may help teachers interpret these changes by articulating a shared vision, providing individualized support, and encouraging professional learning.

Constructivist teaching beliefs (CT) reflect a learner-centered orientation toward teaching and learning. Constructivist pedagogy emphasizes attention to learners and their existing understandings, opportunities for learners to actively examine and develop their understandings, and classroom interaction that supports the development of shared meaning (Richardson, 2003). Consistent with this orientation, CT emphasizes active knowledge construction, meaningful classroom interaction, and responsiveness to students’ ideas and individual needs (Hong et al., 2019; Sharkey & Gash, 2020). Previous studies have linked CT to teachers’ approaches to technology integration, especially when digital tools are used to support student-centered instruction and active learning (Ertmer & Ottenbreit-Leftwich, 2010; Petko, 2012). Recent research has extended this relationship to educational AI, showing that stronger constructivist beliefs are associated with greater acceptance and integration of educational AI and with more positive evaluations of the instructional potential of generative AI (Choi et al., 2023; Cabellos et al., 2024; de Aldama et al., 2026). These findings indicate that pedagogical beliefs are relevant to how teachers judge the instructional value of AI and its compatibility with their teaching goals. Existing AI research, however, has mainly examined CT in relation to technology acceptance, AI integration, or perceptions of instructional potential. Less is known about how CT is associated with leadership context and teachers’ willingness to develop AI competence, or how CT is configured together with TW and BI as components of teachers’ readiness for AI integration, particularly among in-service K-12 teachers.

Teachers’ willingness to develop AI competence (TW) reflects teachers’ orientation toward continued learning and engagement with AI in educational contexts. As AI technologies continue to evolve, teachers are increasingly expected to update their knowledge and skills and critically engage with emerging applications in order to make pedagogically appropriate use of AI tools. Related work on teachers’ AI competence has highlighted the importance of continued professional learning for the development of AI-related knowledge and skills (Delcker et al., 2025). In the present study, TW is treated as a motivational factor associated with teachers’ engagement in AI-related learning and competence development. Despite its potential relevance, empirical evidence remains limited regarding how TW is associated with BI and how it relates to leadership and pedagogical beliefs.

### 2.3. Theoretical Perspectives

#### 2.3.1. Application of UTAUT2 in Educational Technology Research

The Unified Theory of Acceptance and Use of Technology 2 (UTAUT2) is one of the most widely applied frameworks for understanding technology adoption behavior. Building on the original UTAUT model, UTAUT2 incorporates additional constructs, including hedonic motivation, price value, and habit, while retaining core dimensions such as performance expectancy, effort expectancy, facilitating conditions, and social influence (Venkatesh et al., 2012). The framework has been applied across diverse contexts of technology adoption (Tamilmani et al., 2021) and has also been used in educational research to examine teachers’ intentions to use online learning systems, mobile applications, and generative AI tools (Hu et al., 2025; Kong et al., 2024).

In educational technology research, UTAUT2 provides a useful perspective for understanding behavioral intention in relation to the beliefs and conditions surrounding technology use. In the present study, BI is situated within this technology-adoption perspective, while TL is considered a leadership-related contextual factor associated with teachers’ AI adoption intentions.

#### 2.3.2. Application of Self-Determination Theory in Educational Technology Research

Self-Determination Theory (SDT) provides a complementary perspective for understanding the development and internalization of motivation. According to SDT, external regulations may gradually become internalized into more autonomous forms of motivation when individuals’ needs for autonomy, competence, and relatedness are supported by their social environment (Ryan & Deci, 2020). In educational technology research, SDT has been used to explain motivational processes underlying teachers’ engagement in professional learning and development activities (Chiu et al., 2021). It therefore offers a useful lens for understanding teachers’ willingness to develop AI competence as a motivational condition associated with learning and adaptation in response to emerging educational technologies.

In this study, SDT is used to understand TW as a motivational orientation toward continued AI-related learning and competence development.

### 2.4. Research Approaches to Teachers’ Technology Adoption and Behavioral Intention

Research on teachers’ technology adoption and behavioral intention has primarily relied on variable-centered approaches, which examine relationships among constructs across samples (Hu et al., 2025; Hosseini, 2025; Kong et al., 2024). Such approaches are well suited to estimating relationships among variables, but provide less information about whether different combinations of readiness-related characteristics occur among teachers. Person-centered research has increasingly addressed this issue. Earlier studies identified heterogeneity in teachers’ ICT-related dispositions and technology-use motivation (Wu et al., 2021; Xu, 2022), while subsequent work examined profiles involving broader dimensions of technology readiness, including self-efficacy, perceived support, and online teaching practice (Scherer et al., 2021; Paetsch & Franz, 2025).

Person-centered research in AI-related contexts remains more limited. Existing studies have examined AI-acceptance profiles among pre-service teachers (Zhang et al., 2025), AI-readiness profiles among pre-service and in-service foreign-language teachers with behavioral intention examined as an outcome (Liang et al., 2025), and profiles of generative-AI perceptions among foreign-language teachers in relation to continued-use intention (Zang et al., 2026). These studies have advanced understanding of heterogeneity in teachers’ AI acceptance and readiness, but the profile indicators have mainly concerned technology-acceptance beliefs, AI readiness, competence, support, or perceptions of AI. Less is known about how pedagogical beliefs, motivation to develop AI competence, and behavioral intention are configured together among general in-service K-12 teachers. The role of leadership-related conditions in distinguishing such readiness configurations has also received limited attention.

### 2.5. Present Study

The present study draws on selected elements of UTAUT2 and SDT that are directly relevant to the focal constructs and proposed associations. Accordingly, their role is selective and interpretive: UTAUT2 informs the positioning of BI within technology-adoption research, and SDT informs the motivational interpretation of TW; the study does not test either framework in its entirety. The reviewed literature highlights two central issues concerning the associations of TL, CT, and TW with BI and the possibility of distinct readiness profiles based on CT, TW, and BI.

## 3. Theoretical Perspectives and Hypotheses

Teachers’ decisions about using AI are made within school environments where expectations concerning pedagogical use, assessment, bias, privacy, and institutional practice are still developing (Kasneci et al., 2023; Yan et al., 2024). TL may be particularly relevant under these conditions because school leaders can articulate a shared direction, provide individualized support, and encourage professional learning. From the technology-adoption perspective outlined above, these leadership-related conditions may be associated with BI.

Supportive social environments can facilitate the internalization of professional goals and the development of more autonomous forms of motivation (Deci & Ryan, 1985; Ryan & Deci, 2017). For teachers working with emerging AI technologies, leadership support for professional learning may be associated with a stronger TW. TW reflects teachers’ interest in learning about, testing, and critically engaging with AI, and this motivational orientation may in turn be associated with stronger intentions to use AI in teaching.

Pedagogical beliefs may also shape how teachers respond to AI-related instructional change. CT beliefs emphasize active knowledge construction, learner participation, and responsiveness to students’ ideas and individual needs (Richardson, 2003; Hong et al., 2019; Sharkey & Gash, 2020). They have also been associated with more student-centered approaches to technology integration (Ertmer & Ottenbreit-Leftwich, 2010; Petko, 2012). Leadership practices that encourage professional growth and instructional reflection may be associated with teachers’ responses to pedagogical change, while stronger constructivist beliefs may be linked to greater willingness to explore the instructional use of AI. This provides a basis for examining whether TL is indirectly associated with BI through CT and TW in sequence. The following hypotheses were proposed for the variable-centered analysis:

**H1.**  *Transformational leadership is positively associated with teachers’ behavioral intention to use AI tools.*

**H2.**  *Transformational leadership is indirectly associated with teachers’ behavioral intention to use AI tools through teachers’ willingness to develop AI competence.*

**H3.**  *Transformational leadership is sequentially and indirectly associated with teachers’ behavioral intention to use AI tools through constructivist teaching beliefs and teachers’ willingness to develop AI competence.*

## 4. Methods and Measurements

### 4.1. Participants and Procedure

Data were collected in October 2025 through the Wenjuanxing online survey platform among K-12 teachers in mainland China. A convenience sampling approach was adopted. Participation was voluntary and anonymous. A total of 626 questionnaires were returned. Responses completed in less than 120 s were excluded. The cutoff was established based on pilot testing and an estimate of the minimum plausible time required to read and respond to the demographic items and the 29 focal scale items with adequate attention. Cases exhibiting straightlining patterns or insufficient response variability across questionnaire items were also excluded. After excluding 30 invalid responses based on these criteria, 596 valid responses were retained for analysis.

Among the participants, 71.3% were female and 28.7% were male. Regarding age, 3.4% were aged 25 years or younger, 15.8% were aged 26–35 years, 31.5% were aged 36–45 years, 35.7% were aged 46–55 years, and 13.6% were aged 56 years or above. In terms of teaching experience, 8.2% had 5 years or less of teaching experience, 22.1% had 6–10 years, 29.4% had 11–15 years, 30.2% had 16–20 years, and 10.1% had more than 20 years. Regarding teaching level, 51.7% taught in primary schools, 35.7% in junior secondary schools, and 12.6% in senior secondary schools. School location was classified as urban areas, county seats, towns or townships, and rural areas. County seats referred to schools located in the administrative center of a county, whereas towns or townships referred to schools located in township-level localities outside the county seat. 65.9% of the participants worked in urban areas, 10.4% in county seats, 15.8% in towns or townships, and 7.9% in rural areas. Regarding educational attainment, 4.4% held an associate degree or below, 83.6% held a bachelor’s degree, and 12.1% held a master’s degree or above.

### 4.2. Measurements

All questionnaires used in this study were translated into Chinese before being administered to Chinese teachers. For scales originally developed in English, a translation and back translation procedure was adopted to support linguistic and conceptual equivalence. Two researchers with backgrounds in educational technology independently translated the items into Chinese, and a third researcher translated the Chinese version back into English. The research team then compared the original, translated, and back translated versions and discussed any discrepancies to ensure consistency in meaning and conceptual interpretation. Experts in the relevant field were also invited to review the appropriateness and clarity of the items, and the final version was revised based on their feedback. All instruments were rated on a Likert scale ranging from 1 (strongly disagree) to 5 (strongly agree). The results of the confirmatory factor analysis, reliability, and validity tests are reported in Table 1, and the full questionnaire items are presented in Appendix A.

Four constructs were measured in this study. TL was measured using 7 items from Carless et al. (2000), assessing teachers’ perceptions of school leadership behaviors such as vision articulation, individualized consideration, and supportive encouragement. A sample item is “The school leader communicates a clear and positive vision of the future.” CT was measured using 12 items from Teo and Chai (2008), assessing teachers’ constructivist pedagogical beliefs concerning student participation and interaction, active knowledge construction, and responsiveness to students’ ideas and individual needs. A sample item is “The ideas of students are important and should be carefully considered.” In the present study, the 12 items formed three related first-order dimensions, which were labeled Student Interaction, Student-Centered Orientation, and Affective Understanding according to their item content. This three-dimensional representation is broadly consistent with Ngan and Hercz’s (2024) conceptualization of constructivism-oriented teaching conceptions as involving teaching-, teacher-, and student-related aspects. The three first-order factors were subsequently specified to load on a higher-order CT factor, with CT1–CT4, CT5–CT8, and CT9–CT12 serving as their respective indicators. TW was assessed using six items from Delcker et al. (2025). The items capture teachers’ interest in learning about, testing, and critically engaging with AI in education, together with their recognition of its future relevance. A sample item is “I am curious to know more about AI in the context of education and training.” BI was measured using the four-item scale reported by An et al. (2023). The items cover teachers’ intention to use AI in future teaching as well as their willingness to learn from others, seek examples of AI applications, and share AI-related teaching resources and experience. A sample item is “I intend to use AI in teaching in the future.” An et al. (2023) specified these four items as indicators of a single Behavioral Intention construct; the instrument was developed from prior research and teacher interviews and was evaluated through content-validity review, exploratory factor analysis, and confirmatory factor analysis. In the present sample, the four items also showed strong measurement properties (standardized loadings = 0.842–0.953, α = 0.950, CR = 0.952, AVE = 0.833; Table 1), supporting their treatment as a single scale in the analyses.

### 4.3. Data Analysis

Data analyses were conducted in R 4.5.0. CFA and SEM were performed using the lavaan package (Version 0.6-21), and LPA was conducted using the mclust (Version 6.1.2) and tidyLPA (Version 2.0.2) packages. Descriptive statistics and bivariate correlations were first calculated for all study variables. Potential common method variance was assessed at the item level using Harman’s single-factor test and a one-factor CFA. For the Harman test, the 29 focal items were entered into an unrotated principal-axis factor analysis. The CFA specified all 29 items as indicators of a single latent factor. SEM was then used to examine the associations among TL, CT, TW, and BI, including the indirect association from TL to BI via TW and the sequential indirect association from TL to BI via CT and TW. Model fit and structural estimates were obtained using MLR. Because nonparametric bootstrap confidence intervals are not available with MLR in lavaan, indirect associations were evaluated in an otherwise identical model estimated with ML using 5000 bootstrap resamples.

LPA was conducted using standardized composite scores of CT, TW, and BI as indicators of teachers’ readiness for AI integration. For each construct, a composite score was calculated by averaging the corresponding items and was then standardized as a z-score before model estimation. The LPA models estimated profile-specific means, with indicator variances constrained to be equal across profiles and within-profile covariances fixed to zero. Models with varying numbers of profiles were compared using the AIC, BIC, sample-size-adjusted BIC (aBIC), entropy, BLRT, profile size, and theoretical interpretability (Hickendorff et al., 2018; Jung & Wickrama, 2008; Nylund et al., 2007). After the final profile solution was selected, teachers were assigned to their most likely profile based on posterior probabilities. Differences in background characteristics across profiles were examined using chi-square tests, with Fisher’s exact test or Monte Carlo simulated *p*-values used when expected cell counts were small. The background variables included gender, age, teaching experience, teaching level, area, and educational attainment. Multinomial logistic regression was subsequently used to examine the association between TL and profile membership. Two models were estimated: an unadjusted model including TL and an area-adjusted model including TL and area.

## 5. Results

The measurement model specified TL, TW, and BI as first-order latent factors and CT as a second-order latent factor comprising three first-order dimensions, with no correlated item residuals specified. The CFA indicated an acceptable model fit, *χ*^2^(368) = 1127.01, robust CFI = 0.939, robust TLI = 0.933, robust RMSEA = 0.076, 90% CI [0.071, 0.081], and SRMR = 0.048. Standardized item loadings ranged from 0.738 to 0.953 (Table 1).

Harman’s single-factor analysis showed that the first factor accounted for 49.9% of the variance across the 29 focal items, close to the 50% reference value commonly used in applications of the test (Fuller et al., 2016). A single-factor CFA showed poor fit, *χ*^2^(377) = 11,391.74, CFI = 0.479, TLI = 0.439, RMSEA = 0.222, and SRMR = 0.165, providing little support for a single-factor representation of the item covariance.

### 5.1. Descriptive Statistics and Bivariate Correlations

Table 2 presents the descriptive statistics, normality indices, and bivariate correlations among the study variables. Mean scores for the four core variables ranged from 3.99 to 4.44, with standard deviations ranging from 0.57 to 0.78. All skewness and kurtosis values were within recommended thresholds (|skewness| < 2, |kurtosis| < 7), indicating no substantial departures from normality. TL, CT, TW, and BI were all positively correlated, with correlation coefficients ranging from 0.410 to 0.665 (all *p*s < 0.01). Multicollinearity diagnostics indicated no evidence of problematic collinearity among the core variables, with VIF values ranging from 1.26 to 1.80, well below the commonly used threshold of 5.

### 5.2. Structural Equation Modeling

**Figure 1:**
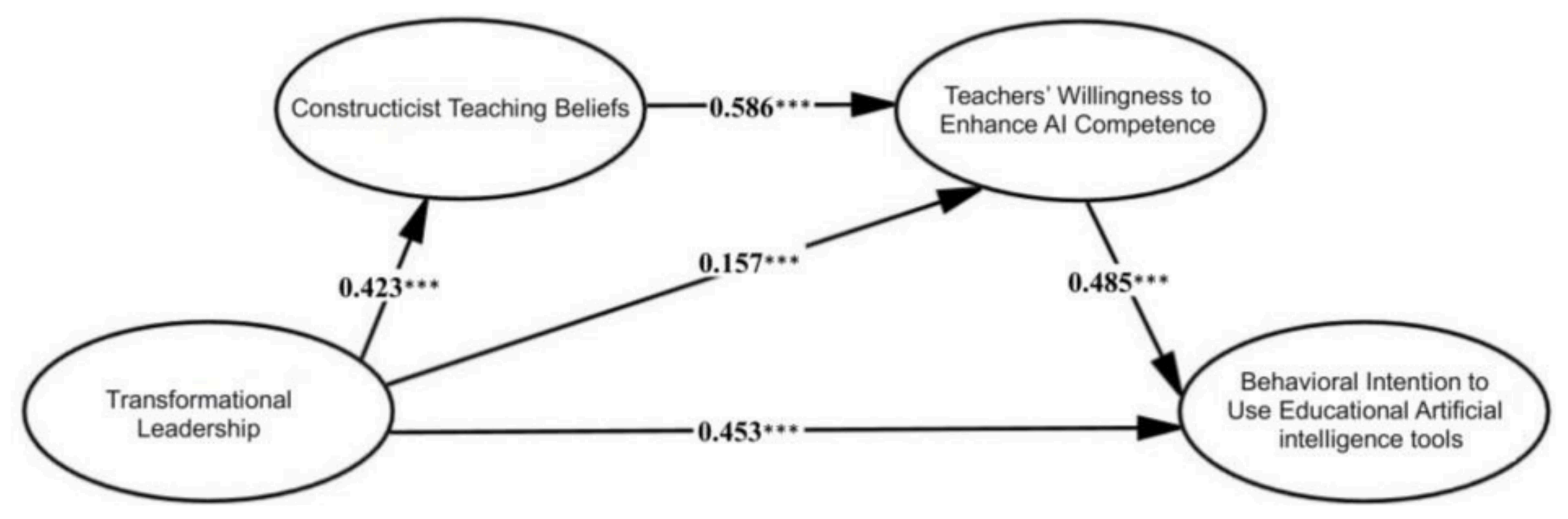
Structural model of the associations among TL, CT, TW, and BI. Note. *** *p* < 0.001.

The structural model exhibited acceptable fit, scaled *χ*^2^(369) = 1128.46, robust CFI = 0.939, robust TLI = 0.933, robust RMSEA = 0.076, 90% CI [0.071, 0.081], and SRMR = 0.050. As shown in Figure 1 and Table 3, TL was positively associated with BI (β = 0.453, *p* < 0.001), supporting H1. The constituent paths of the hypothesized indirect associations were also significant: TL was positively associated with TW (β = 0.157, *p* < 0.001), TW was positively associated with BI (β = 0.485, *p* < 0.001), TL was positively associated with CT (β = 0.423, *p* < 0.001), and CT was positively associated with TW (β = 0.586, *p* < 0.001).

Bootstrap analyses based on 5000 resamples showed a significant indirect association between TL and BI through TW (β = 0.076, 95% CI [0.036, 0.118]), supporting H2. A significant sequential indirect association involving CT and TW was also observed (β = 0.120, 95% CI [0.082, 0.159]), supporting H3. Although the sequential indirect association was numerically larger than the indirect association through TW alone, the difference between the two estimates was not statistically significant (Δβ = 0.044, 95% CI [−0.021, 0.110]). The total indirect association was 0.196 (95% CI [0.151, 0.243]), whereas the total association between TL and BI was 0.650 (95% CI [0.576, 0.712]). The model accounted for 61.9% of the variance in BI (R^2^ = 0.619).

### 5.3. Latent Profile Analysis

#### 5.3.1. Profile Identification

Latent profile analysis (LPA) was conducted to examine heterogeneity among teachers in CT, TW, and BI. Mean scores for CT, TW, and BI were standardized as z-scores and used as profile indicators. As shown in Table 4, model selection was based on statistical fit, classification accuracy, profile size, and substantive interpretability. Although the five-profile solution yielded the lowest AIC and slightly higher entropy, it was not selected because it had higher BIC and adjusted BIC values, and the BLRT did not indicate a significant improvement over the four-profile solution (*p* = 0.099). In contrast, the four-profile solution showed adequate classification accuracy (entropy = 0.870), all profiles exceeded the minimum size criterion, and the BLRT supported improvement over the three-profile solution (*p* < 0.01). Substantively, the four-profile solution provided the most interpretable distinctions across CT, TW, and BI and was retained for subsequent analyses.

The average posterior probabilities (AvePP) further supported the adequacy of the classification (see Table 5). In the four-profile solution, diagonal AvePP values ranged from 0.829 to 0.991, exceeding the recommended threshold of 0.80 and indicating satisfactory classification accuracy.

#### 5.3.2. Interpretation of Teachers’ Latent Profiles

**Figure 2:**
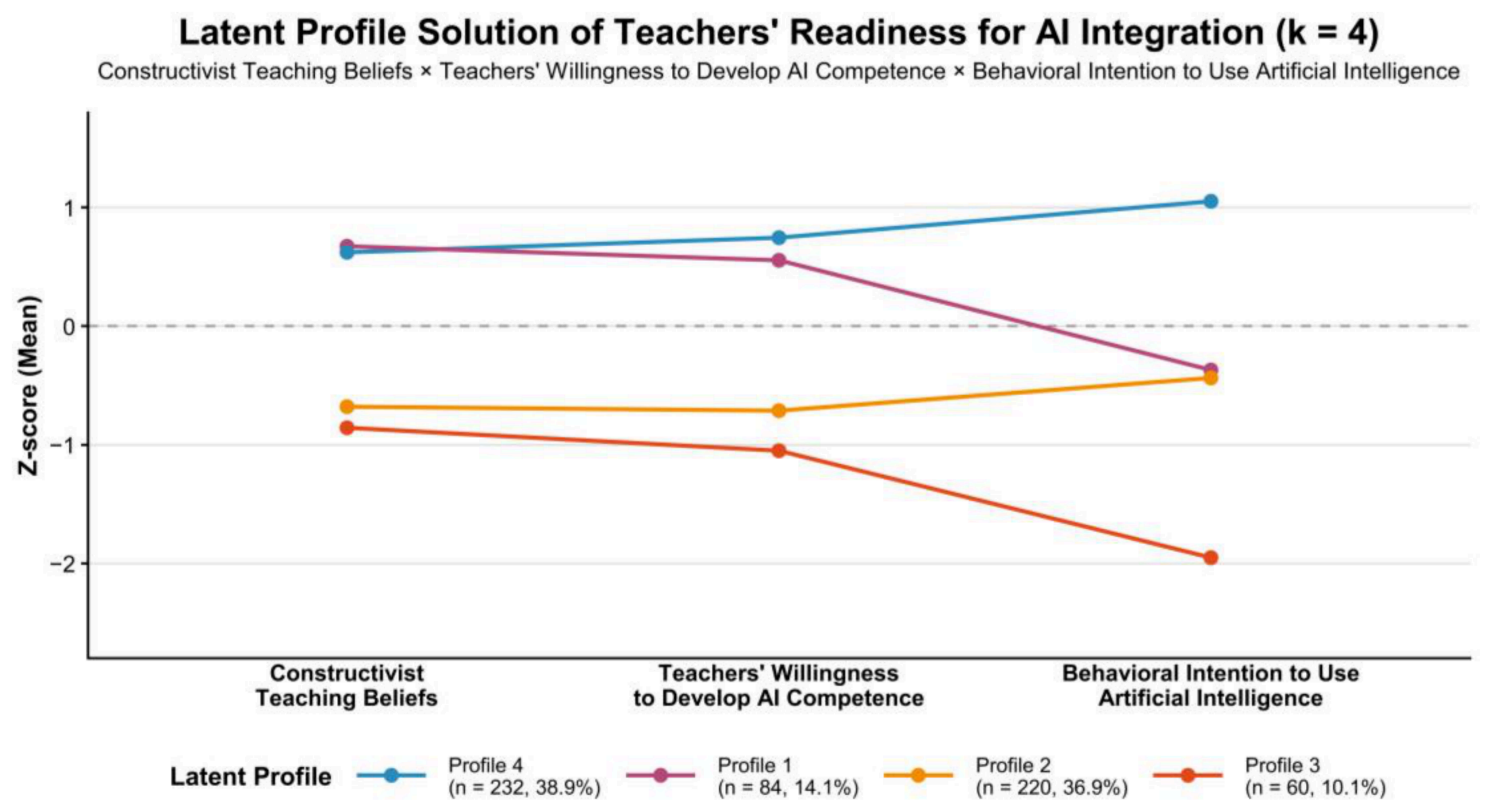
Standardized indicator means across the four latent profiles.

The four-profile solution identified through latent profile analysis revealed heterogeneity in teachers’ readiness for AI integration as reflected in CT, TW, and BI (see Figure 2 and Table 6). Table 6 presents the standardized scores (z values) of the profile indicators, together with the defining characteristics and labels of the four profiles. The profile labels were assigned descriptively based on the relative levels of CT, TW, and BI within each profile. The Favorable Orientation but Behavioral Hesitation Profile (n = 84, 14.1%) was characterized by above-average scores for CT (z = 0.673) and TW (z = 0.555), together with a below-average score for BI (z = −0.369). The Limited Orientation and Behavioral Hesitation Profile (n = 220, 36.9%) showed below-average scores across all three indicators (CT: z = −0.680; TW: z = −0.712; BI: z = −0.437), suggesting a below-average pattern that was less pronounced than that of Profile 3. The Low Orientation and Weak Behavioral Inclination Profile (n = 60, 10.1%) was marked by consistently low scores across all three indicators (CT: z = −0.858; TW: z = −1.050; BI: z = −1.952), with BI markedly lower than the other two indicators. By contrast, the Favorable Orientation and Behavioral Inclination Profile (n = 232, 38.9%) showed above-average scores across all three indicators (CT: z = 0.623; TW: z = 0.746; BI: z = 1.053), indicating a consistently higher-than-average pattern of constructivist beliefs, willingness to develop AI competence, and behavioral intention to use AI tools.

#### 5.3.3. Demographic Differences Across Profiles

To examine whether profile membership was associated with teacher and school background characteristics, the distributions of these characteristics were compared across the four profiles (see Table 7). Chi-square tests showed that gender (*χ*^2^ = 2.59, *df* = 3, *p* = 0.459), age group (*χ*^2^ = 15.63, *df* = 12, *p* = 0.209), years of teaching experience (*χ*^2^ = 20.38, *df* = 12, *p* = 0.060), and educational attainment (*χ*^2^ = 6.63, *df* = 6, *p* = 0.357) were not significantly associated with profile membership. By contrast, teaching level (*χ*^2^ = 14.66, *df* = 6, *p* = 0.023) and area (Fisher’s exact test, *p* < 0.001) were significantly associated with profile membership. Given the overlap between teaching level and area in the sample, an additional model comparison was conducted to examine whether teaching level provided incremental explanatory value beyond area. Adding teaching level to the model did not significantly improve model fit after area had been included (Δ*χ*^2^ = 7.50, Δ*df* = 6, *p* = 0.277). Therefore, area was included in the adjusted multinomial regression model.

#### 5.3.4. Associations Between Transformational Leadership and Profile Membership

Multinomial logistic regression was conducted to examine the association between TL and teachers’ profile membership, using the Favorable Orientation but Behavioral Hesitation Profile (Profile 1) as the reference category. As shown in Table 8, the unadjusted model (Model 1) indicated that TL did not significantly differentiate teachers in Profile 2 from those in Profile 1 (OR = 0.89, 95% CI [0.66, 1.20], *p* = 0.452). In contrast, higher TL was associated with lower odds of membership in Profile 3 (OR = 0.33, 95% CI [0.22, 0.51], *p* < 0.001) and higher odds of membership in Profile 4 (OR = 4.69, 95% CI [3.28, 6.70], *p* < 0.001), relative to Profile 1.

Area was subsequently included in Model 2 to examine whether the association between TL and profile membership remained after accounting for area. A likelihood-ratio test indicated that the area-adjusted model provided a significantly better fit than the unadjusted model (Δ*χ*^2^ = 25.70, Δ*df* = 9, *p* = 0.002). The pattern of associations remained largely unchanged: TL was not significantly associated with membership in Profile 2 relative to Profile 1 (OR = 0.85, 95% CI [0.63, 1.16], *p* = 0.310), whereas higher TL was associated with lower odds of membership in Profile 3 (OR = 0.29, 95% CI [0.18, 0.46], *p* < 0.001) and higher odds of membership in Profile 4 (OR = 4.67, 95% CI [3.22, 6.73], *p* < 0.001). Compared with teachers in urban areas, teachers in towns or townships had lower odds of membership in Profile 2 (OR = 0.44, 95% CI [0.23, 0.85], *p* = 0.014) and Profile 3 (OR = 0.28, 95% CI [0.09, 0.85], *p* = 0.025), relative to Profile 1.

## 6. Discussion

### 6.1. Transformational Leadership and Teachers’ Behavioral Intention to Use AI Tools: Direct and Indirect Associations

The variable-centered results showed that TL was positively associated with BI (β = 0.453, *p* < 0.001), which is consistent with prior research linking TL to teachers’ technology integration (Schmitz et al., 2023) and with AI adoption research showing that school- and teacher-level factors are related to teachers’ intention to use AI (Wu et al., 2025). In the context of generative AI, where pedagogical norms, assessment practices, and institutional expectations are still developing, TL may reflect leadership practices that provide signals about the appropriateness and value of AI use within the school. This interpretation is consistent with UTAUT2’s broader emphasis on supportive conditions surrounding technology use (Venkatesh et al., 2012).

The significant indirect association through TW showed that higher perceived TL was associated with greater willingness to develop AI competence, which was in turn associated with stronger BI (β = 0.076). Research indicates that teachers’ motivation to engage with AI-related learning is related to expectancy beliefs, perceived usefulness, and the perceived costs associated with technological adaptation (Bozer Özsaraç & Ergin, 2025). This pattern is consistent with the possibility that leadership practices supporting professional learning and innovation are relevant to teachers’ motivation to invest time and effort in AI-related competence development, particularly when such learning is perceived as valuable and manageable. The sequential indirect association involving TL, CT, TW, and BI was also significant (β = 0.120). Within the specified model, higher perceived TL was associated with stronger CT, stronger CT was associated with greater TW, and greater TW was associated with stronger BI. Constructivist beliefs may be relevant to this pattern because teachers who place greater emphasis on active knowledge construction, learner participation, and student-centered instruction may be more inclined to explore how emerging technologies can support their pedagogical goals (Ertmer & Ottenbreit-Leftwich, 2010; Petko, 2012). Such pedagogical orientations may provide a stronger basis for continued engagement with AI-related learning and competence development. Although the sequential indirect association was numerically larger than the indirect association through TW alone, the bootstrap comparison showed that the difference was not statistically significant (Δβ = 0.044, 95% CI [−0.021, 0.110]). The person-centered results further showed that the associations identified in the variable-centered analysis did not correspond to a single pattern across teachers, as CT, TW, and BI were combined differently across the identified profiles.

### 6.2. Heterogeneity in Teachers’ Readiness for AI Integration

Recent person-centered research has documented heterogeneity in teachers’ technology readiness, with profiles defined by technology-related dispositions, motivation, self-efficacy, perceived support, and teaching practice (Wu et al., 2021; Xu, 2022; Scherer et al., 2021; Paetsch & Franz, 2025). AI-focused profile studies have extended this work to AI acceptance and readiness, although current evidence has largely come from pre-service teachers or specialized language-teacher samples and has centered on technology-acceptance beliefs, AI readiness, competence, support, or perceptions of AI (Zhang et al., 2025; Liang et al., 2025; Zang et al., 2026). The present study extends this research to general in-service K-12 teachers by examining the joint configuration of CT, TW, and BI.

The Favorable Orientation but Behavioral Hesitation Profile (Profile 1; 14.1%) was characterized by above-average CT and TW and below-average BI. Its distinctiveness becomes clearer in comparison with Profile 4. Both profiles showed relatively high CT and TW, whereas BI was much lower in Profile 1. Teachers in Profile 1 therefore held relatively favorable constructivist teaching beliefs and expressed greater willingness to develop AI competence, while remaining comparatively hesitant about using AI in teaching. Such hesitation may be related to considerations that were not captured by CT or TW. Research on educational applications of large language models has identified concerns about technological readiness, model performance and reliability, privacy, transparency, and responsible implementation (Kasneci et al., 2023; Yan et al., 2024). These factors were not measured in the present study, so their relevance to Profile 1 requires further examination.

The multinomial logistic regression results further showed that TL was associated with teachers’ profile membership. In the area-adjusted model, higher TL was associated with lower odds of membership in the Low Orientation and Weak Behavioral Inclination Profile relative to the Favorable Orientation but Behavioral Hesitation Profile (Profile 3 vs. 1: OR = 0.29, *p* < 0.001) and higher odds of membership in the Favorable Orientation and Behavioral Inclination Profile relative to Profile 1 (Profile 4 vs. 1: OR = 4.67, *p* < 0.001). By contrast, TL did not significantly differentiate the Limited Orientation and Behavioral Hesitation Profile from Profile 1 (Profile 2 vs. 1: OR = 0.85, *p* = 0.310). This pattern is consistent with prior research linking transformational leadership to teachers’ technology integration and related professional practices (Schmitz et al., 2023, 2025). One possible interpretation is that higher perceived TL may reflect school leadership conditions characterized by clearer direction for instructional change, stronger support for professional learning, and greater encouragement for experimentation with emerging technologies. Such leadership practices may be particularly relevant when teachers are required to make sense of rapidly changing AI applications and uncertain expectations for classroom use. This interpretation is consistent with the observed pattern in which higher TL was associated with greater odds of membership in Profile 4 and lower odds of membership in Profile 3 relative to Profile 1. The nonsignificant contrast between Profiles 2 and 1, however, indicates that TL did not differentiate all forms of behavioral hesitation. Teachers in these two profiles reported similarly low BI despite differing substantially in CT and TW, suggesting that other AI-related beliefs, concerns, or opportunities for instructional use may also be relevant.

Profile membership also differed across school locations. Compared with teachers in urban areas, those in towns or townships had lower odds of belonging to Profiles 2 and 3 relative to Profile 1. Research in resource-constrained school contexts has documented barriers involving infrastructure, connectivity, device access, professional development, and institutional support in teachers’ engagement with AI-related technologies (Mokoena & Seeletse, 2025). These findings illustrate how local conditions may be relevant to teachers’ opportunities to engage with educational AI. The present study did not directly measure these conditions, so the factors underlying the observed school-location differences remain to be examined.

### 6.3. Theoretical Implications

The variable-centered findings extend research on teachers’ AI adoption by examining leadership context, constructivist teaching beliefs, willingness to develop AI competence, and behavioral intention within the same model. UTAUT2 situates BI within a technology-adoption perspective, SDT informs the interpretation of TW as a willingness to invest in continued competence development, and constructivist pedagogy provides the conceptual basis for CT. The observed associations connect school leadership with teachers’ pedagogical beliefs and willingness to develop AI competence, while TW is further associated with teachers’ intention to use AI in teaching. This extends the scope of AI-adoption research by incorporating leadership and teacher-level pedagogical and motivational factors into the analysis of behavioral intention.

The person-centered findings extend research on teachers’ AI readiness in terms of both the population examined and the components used to characterize readiness. Existing AI-focused profile studies have mainly examined pre-service teachers or specialized language-teacher groups and have defined profiles primarily through technology-acceptance beliefs, AI readiness, competence, support, or perceptions of AI. By focusing on general in-service K-12 teachers and jointly modeling CT, TW, and BI, the present study brings pedagogical beliefs, motivation for continued AI competence development, and behavioral intention into the same readiness configuration. The resulting profiles show that these components do not vary uniformly across teachers, as relatively favorable CT and TW can coexist with substantially different levels of BI. This extends the conceptualization of AI readiness beyond technology-related competence and acceptance to include how pedagogical, motivational, and behavioral components are configured within teachers.

### 6.4. Practical Implications

The findings offer practical implications for school leaders and policymakers seeking to support AI integration in K-12 education. Efforts to strengthen teachers’ behavioral intention to use AI tools should go beyond technical training and infrastructure. School leaders may need to articulate a clear educational vision for AI use, provide individualized support, and create school conditions that encourage instructional experimentation and professional learning. Professional development may also be more effective when AI-related training is connected to broader instructional goals and pedagogical values rather than limited to tool operation. The profile results further suggest that support should be differentiated across teacher groups. Because TL did not distinguish all readiness profiles, teachers’ AI-related self-efficacy, concerns, and opportunities for classroom-based practice may also require attention. In addition, the observed area differences indicate that the relevance of local context should be considered when designing AI-related professional support.

### 6.5. Limitations and Future Directions

Despite its theoretical and practical contributions, this study has several limitations. One limitation concerns the cross-sectional and self-reported nature of the data. The cross-sectional design limits the interpretation of the structural associations. Because TL, CT, TW, and BI were measured at the same time, the findings do not establish causal relations or the temporal ordering implied by the specified indirect associations. Longitudinal studies are needed to examine whether these associations persist over time and whether the proposed ordering is supported across measurement occasions. In addition, all focal variables were assessed through teacher self-reports collected at the same time. Although the common-method diagnostics did not support a single-factor representation of the item covariance, common method variance and social desirability effects cannot be entirely ruled out. Future research could reduce this concern through temporally separated measurement and multiple data sources, such as school leaders, classroom observations, or behavioral indicators.

Another limitation concerns the measurement structure of CT. The higher-order representation of CT was supported by comparisons with alternative measurement specifications (Appendix A Table A2). The main structural associations remained substantively similar when CT was represented using a reliability-corrected composite score (Appendix A Table A3). Future research using independent samples would help further establish the stability of this measurement structure.

A further limitation concerns the sampling approach and geographic representation. Participants were recruited through convenience sampling, and the sample was concentrated in urban schools (65.9%), with teachers from rural schools accounting for 7.9% of the participants. This uneven distribution narrows the basis for generalizing the findings across K-12 teachers in different parts of mainland China and may also reduce the precision of comparisons across school locations. Local differences in educational infrastructure, access to digital technologies, professional learning opportunities, and school-level support may be relevant to the observed profile differences. Future studies using geographically stratified or probability-based samples, together with direct measures of these local conditions, would provide a stronger basis for examining the stability and contextual meaning of area-related differences in readiness profiles.

Another limitation relates to the outcome variable and the scope of variables included in the model. BI is an important precursor to technology adoption, but it does not necessarily reflect teachers’ actual use of AI in the classroom. The study also did not directly examine why favorable CT and TW do not always coincide with stronger BI. The Favorable Orientation but Behavioral Hesitation Profile suggests that some teachers may hold favorable constructivist beliefs and willingness to develop AI competence while still reporting lower BI. This pattern points to the possible relevance of additional factors, such as AI-related self-efficacy, perceived risks, ethical and privacy concerns, and technology-related anxiety. Future research could incorporate actual-use indicators and examine these additional factors to clarify how teachers’ pedagogical beliefs and willingness to develop AI competence are related to the integration of AI into teaching.

## 7. Conclusions

Drawing on complementary perspectives from UTAUT2 and SDT, this study examined BI and teachers’ readiness for AI integration among 596 K-12 teachers in mainland China. The findings showed that TL was positively associated with BI and was also indirectly associated with BI through TW and through the sequential association involving CT and TW. The person-centered analysis further identified four readiness profiles characterized by different configurations of CT, TW, and BI. In particular, favorable CT and TW were observed alongside different levels of BI, highlighting variation in teachers’ readiness for AI integration. Overall, the findings indicate the relevance of leadership context, pedagogical beliefs, and willingness to develop AI competence for understanding teachers’ intentions and readiness for AI integration.

## Figures and Tables

**Table 1:** Standardized factor loadings, reliability, and convergent validity indices.

Construct	Number of Items	λ Range	α	CR	AVE
TL	7	0.811–0.951	0.970	0.969	0.819
CT	12	0.738–0.938	0.961	0.956	0.647
TW	6	0.754–0.950	0.935	0.936	0.712
BI	4	0.842–0.953	0.950	0.952	0.833

Note. λ = standardized item loading; α = Cronbach’s alpha; CR = composite reliability; AVE = average variance extracted. For CT, λ refers to item loadings on the three first-order factors; CR and AVE refer to the higher-order CT construct.

**Table 2:** Descriptive Statistics and Bivariate Correlations of Core Variables.

Variable	Mean	SD	Skewness	Kurtosis	1	2	3	4
1. CT	4.44	0.571	−1.39	4.48				
2. TW	4.31	0.619	−0.70	0.88	0.647 **			
3. TL	3.99	0.779	−0.51	0.16	0.410 **	0.414 **		
4. BI	4.23	0.664	−0.66	1.06	0.537 **	0.665 **	0.640 **	

Note. *N* = 596, ** *p* < 0.01.

**Table 3:** Standardized Path Coefficients and Bootstrap Confidence Intervals.

Association	β	SE	z	*p*	95% CI
Direct Association					
TL → CT	0.423	0.051	8.23	<0.001	[0.317, 0.518]
TL → TW	0.157	0.041	3.81	<0.001	[0.076, 0.237]
CT → TW	0.586	0.045	13.00	<0.001	[0.492, 0.670]
TL → BI	0.453	0.039	11.58	<0.001	[0.378, 0.526]
TW → BI	0.485	0.041	11.87	<0.001	[0.403, 0.561]
Indirect Association					
TL → TW → BI	0.076	0.022	3.40	<0.001	[0.036, 0.118]
TL → CT → TW → BI	0.120	0.022	5.44	<0.001	[0.082, 0.159]
Total Indirect Association	0.196	0.029	6.68	<0.001	[0.151, 0.243]
Total Association	0.650	0.062	10.41	<0.001	[0.576, 0.712]

Note. β = standardized coefficient; SE = standard error; CIs are percentile bootstrap confidence intervals for standardized estimates based on 5000 bootstrap samples.

**Table 4:** Model Fit Indices for Latent Profile Analysis (N = 596).

Profile	LL	npar	AIC	BIC	aBIC	Entropy	pBLRT	Min Class %
1	−2535.6	6	5083.1	5109.5	5090.4	1.000	—	100.0
2	−2264.4	10	4548.7	4592.6	4560.9	0.812	<0.01	49.5
3	−2159.8	14	4347.5	4409.0	4364.5	0.893	<0.01	7.6
4	−2095.9	18	4227.8	4306.9	4249.7	0.870	<0.01	10.1
5	−2091.8	22	4227.6	4324.2	4254.4	0.892	0.099	5.5

Note. LL = log-likelihood. AIC = Akaike information criterion; BIC = Bayesian information criterion; aBIC = sample-size-adjusted BIC; BLRT = bootstrap likelihood ratio test.

**Table 5:** Latent Profiles and Classification Accuracy (Average Posterior Probabilities).

Profile	1	2	3	4	*N*	%
1. Favorable Orientation but Behavioral Hesitation Profile	0.829	0.170	0.000	0.001	84	14.1
2. Limited Orientation and Behavioral Hesitation Profile	0.091	0.899	0.006	0.004	220	36.9
3. Low Orientation and Weak Behavioral Inclination Profile	0.001	0.007	0.991	0.000	60	10.1
4. Favorable Orientation and Behavioral Inclination Profile	0.022	0.009	0.000	0.969	232	38.9

**Table 6:** Profile Characteristics Based on Standardized Indicator Scores.

Profile	Profile Size	Profile Indicator Variables		
		CT M (95% CI)	TW M (95% CI)	BI M (95% CI)
Profile 1: Favorable Orientation but Behavioral Hesitation Profile	84 (14.1%)	0.67 (0.58, 0.77)	0.56 (0.42, 0.69)	−0.37 (−0.41, −0.33)
Profile 2: Limited Orientation and Behavioral Hesitation Profile	220 (36.9%)	−0.68 (−0.79, −0.57)	−0.71 (−0.80, −0.63)	−0.44 (−0.47, −0.40)
Profile 3: Low Orientation and Weak Behavioral Inclination Profile	60 (10.1%)	−0.86 (−1.15, −0.57)	−1.05 (−1.32, −0.78)	−1.95 (−2.12, −1.79)
Profile 4: Favorable Orientation and Behavioral Inclination Profile	232 (38.9%)	0.62 (0.55, 0.70)	0.75 (0.67, 0.82)	1.05 (1.02, 1.08)

Note. M = standardized mean (Z-score). 95% CI = 95% confidence interval. All reported means represent profile-specific Z-scores relative to the overall sample (M = 0, SD = 1).

**Table 7:** Demographic Characteristics Across Latent Profiles (*N* = 596).

Characteristic	Profile 1 (*N* = 84)	Profile 2 (*N* = 220)	Profile 3 (*N* = 60)	Profile 4 (*N* = 232)	Test	*p*
Gender, n (%)					*χ*^2^(3) = 2.59	0.459
Male	24 (28.6)	71 (32.3)	17 (28.3)	59 (25.4)		
Female	60 (71.4)	149 (67.7)	43 (71.7)	173 (74.6)		
Age, n (%)					*χ*^2^(12) = 15.63	0.209
≤25	1 (1.2)	10 (4.5)	2 (3.3)	7 (3.0)		
26–35	10 (11.9)	33 (15.0)	10 (16.7)	41 (17.7)		
36–45	31 (36.9)	68 (30.9)	12 (20.0)	77 (33.2)		
46–55	35 (41.7)	75 (34.1)	22 (36.7)	81 (34.9)		
≥56	7 (8.3)	34 (15.5)	14 (23.3)	26 (11.2)		
Years of Teaching Experience, n (%)					*χ*^2^(12) = 20.38	0.060
≤5	6 (7.1)	20 (9.1)	4 (6.7)	19 (8.2)		
6–10 years	16 (19.0)	47 (21.4)	16 (26.7)	53 (22.8)		
11–15 years	33 (39.3)	66 (30.0)	8 (13.3)	68 (29.3)		
16–20 years	24 (28.6)	61 (27.7)	20 (33.3)	75 (32.3)		
>20 years	5 (6.0)	26 (11.8)	12 (20.0)	17 (7.3)		
Teaching level, n (%)					*χ*^2^(6) = 14.66	0.023
Primary	45 (53.6)	99 (45.0)	25 (41.7)	139 (59.9)		
Junior Secondary	27 (32.1)	92 (41.8)	28 (46.7)	66 (28.4)		
Senior Secondary	12 (14.3)	29 (13.2)	7 (11.7)	27 (11.6)		
Area, n (%)					Fisher’s exact	<0.001
Urban	47 (55.9)	150 (68.2)	35 (58.3)	161 (69.4)		
County seat	11 (13.1)	19 (8.6)	7 (11.7)	25 (10.8)		
Town or township	21 (25.0)	30 (13.6)	5 (8.3)	38 (16.4)		
Rural	5 (6.0)	21 (9.5)	13 (21.7)	8 (3.4)		
Educational attainment, n (%)					*χ*^2^(6) = 6.63	0.357
Associate degree or below	2 (2.4)	12 (5.5)	3 (5.0)	9 (3.9)		
Bachelor’s degree	72 (85.7)	178 (80.9)	55 (91.7)	193 (83.2)		
Master’s degree or above	10 (11.9)	30 (13.6)	2 (3.3)	30 (12.9)		

**Table 8:** Multinomial Logistic Regression Results for the Association Between Transformational Leadership and Profile Membership.

Predictor	Profile 2 vs. 1 OR [95% CI]	Profile 3 vs. 1 OR [95% CI]	Profile 4 vs. 1 OR [95% CI]
Model 1: Unadjusted model			
Intercept	2.53 [1.95, 3.30] ***	0.35 [0.22, 0.55] ***	1.88 [1.40, 2.51] ***
TL (Z-score)	0.89 [0.66, 1.20]	0.33 [0.22, 0.51] ***	4.69 [3.28, 6.70] ***
Model 2: Area-adjusted model			
Intercept	3.09 [1.79, 4.08] ***	0.38 [0.21, 0.74] **	2.17 [1.51, 3.12] ***
TL (Z-score)	0.85 [0.63, 1.16]	0.29 [0.18, 0.46] ***	4.67 [3.22, 6.73] ***
Area (Ref. = Urban)			
County seat	0.50 [0.21, 1.15]	0.46 [0.18, 1.81]	1.16 [0.47, 2.82]
Town or Township	0.44 [0.23, 0.85] *	0.28 [0.09, 0.85] *	0.52 [0.26, 1.04]
Rural	1.29 [0.43, 3.46]	3.20 [1.00, 10.30]	0.53 [0.15, 1.86]

Note. * *p* < 0.05, ** *p* < 0.01, *** *p* < 0.001. Reference group = Profile 1 (Favorable Orientation but Behavioral Hesitation Profile).

## Data Availability

The datasets used in the current study are available from the corresponding author upon reasonable request.

## Appendix A

**Table A1:** Questionnaire Items.

Variable	Item Code	Final English Item
TL	TL1	The school leader communicates a clear and positive vision of the future.
	TL2	The school leader treats staff as individuals, supports and encourages their development.
	TL3	The school leader gives encouragement and recognition to staff.
	TL4	The school leader fosters trust, involvement, and cooperation among team members.
	TL5	The school leader encourages thinking about problems in new ways and questions assumptions.
	TL6	The school leader is clear about his/her values and practices what he/she preaches.
	TL7	The school leader instills pride and respect in others and inspires by being highly competent.
CT	CT1	The ideas of students are important and should be carefully considered.
	CT2	Effective teaching encourages more discussion and hands-on activities for students.
	CT3	Students should be given many opportunities to express their ideas.
	CT4	In a good classroom there is a democratic and free atmosphere which stimulates students to think and interact.
	CT5	Every child is unique or special and deserves an education tailored to his or her particular needs.
	CT6	Good teachers always encourage students to think for answers themselves.
	CT7	The focus of teaching is to help students construct knowledge from their learning experience instead of knowledge communication.
	CT8	Different objectives and expectations in learning should be applied to different students.
	CT9	Good teachers always make their students feel important.
	CT10	Instruction should be flexible enough to accommodate individual differences among students.
	CT11	It is important that a teacher understands the feelings of the students.
	CT12	Learning means students have ample opportunities to explore, discuss and express their ideas.
TW	TW1	I am curious to know more about AI in the context of education and training.
	TW2	I want to learn more about AI in the context of education and training.
	TW3	I am interested in testing AI in the context of education and training.
	TW4	I am interested in a critical discussion about AI.
	TW5	I believe AI will play an important role in the future.
	TW6	AI should be an important topic in the future.
BI	BI1	I am willing to learn the experience of AI education application from others.
	BI2	I am willing to learn the case of AI education application from the Internet
	BI3	I am happy to share my AI teaching resources and experience with others.
	BI4	I intend to use AI in teaching in the future.

**Table A2:** Alternative Measurement Specifications for Constructivist Teaching Beliefs.

Model	*χ* ^2^	*df*	CFI	TLI	RMSEA	SRMR
One-factor	567.81	54	0.832	0.795	0.201	0.060
Two-factor: F1 + F2 merged	349.83	53	0.901	0.877	0.156	0.047
Two-factor: F1 + F3 merged	491.50	53	0.865	0.832	0.182	0.056
Two-factor: F2 + F3 merged	261.10	53	0.931	0.915	0.130	0.038
Three correlated factors	100.32	51	0.985	0.981	0.061	0.023

Note. Robust fit indices from MLR estimation are reported. F1 = Student Interaction; F2 = Student-Centered Orientation; F3 = Affective Understanding. The higher-order CT representation has the same CT-only fit as the three-correlated-factor model because a second-order factor defined by exactly three first-order factors is just-identified at the higher-order level. Robust scaled *χ*^2^ difference tests favored the three-factor model over the one-factor model and each two-factor merger (all *p*s < 0.001).

**Table A3:** Sensitivity of Structural Estimates to the Representation of CT.

Structural Association	Higher-Order CT β	Reliability-Corrected Composite CT β
TL → CT	0.423	0.427
TL → TW	0.157	0.157
CT → TW	0.586	0.580
TL → BI	0.453	0.454
TW → BI	0.485	0.485
TL → TW → BI	0.076	0.076
TL → CT → TW → BI	0.120	0.120
Total indirect association	0.196	0.196
Total association	0.650	0.650

Note. CT was re-estimated as a reliability-corrected single-indicator latent variable based on the 12-item composite score. The structural estimates were virtually unchanged. Model-fit indices are not compared across these specifications because the observed-variable representations differ. TW = willingness to develop AI competence; BI = behavioral intention.
